# High Endothelial Venules and Lymphatic Vessels in Tertiary Lymphoid Organs: Characteristics, Functions, and Regulation

**DOI:** 10.3389/fimmu.2016.00491

**Published:** 2016-11-09

**Authors:** Nancy H. Ruddle

**Affiliations:** ^1^Department of Epidemiology of Microbial Diseases, School of Public Health, Yale University School of Medicine, New Haven, CT, USA

**Keywords:** lymph node, lymphatic vessel, high endothelial venule, tertiary lymphoid organ, autoimmunity, inflammation, cancer, lymphotoxin

## Abstract

High endothelial venules (HEVs) and lymphatic vessels (LVs) are essential for the function of the immune system, by providing communication between the body and lymph nodes (LNs), specialized sites of antigen presentation and recognition. HEVs bring in naïve and central memory cells and LVs transport antigen, antigen-presenting cells, and lymphocytes in and out of LNs. Tertiary lymphoid organs (TLOs) are accumulations of lymphoid and stromal cells that arise and organize at ectopic sites in response to chronic inflammation in autoimmunity, microbial infection, graft rejection, and cancer. TLOs are distinguished from primary lymphoid organs – the thymus and bone marrow, and secondary lymphoid organs (SLOs) – the LNs, spleen, and Peyer’s patches, in that they arise in response to inflammatory signals, rather than in ontogeny. TLOs usually do not have a capsule but are rather contained within the confines of another organ. Their structure, cellular composition, chemokine expression, and vascular and stromal support resemble SLOs and are the defining aspects of TLOs. T and B cells, antigen-presenting cells, fibroblast reticular cells, and other stromal cells and vascular elements including HEVs and LVs are all typical components of TLOs. A key question is whether the HEVs and LVs play comparable roles and are regulated similarly to those in LNs. Data are presented that support this concept, especially with regard to TLO HEVs. Emerging data suggest that the functions and regulation of TLO LVs are also similar to those in LNs. These observations support the concept that TLOs are not merely cellular accumulations but are functional entities that provide sites to generate effector cells, and that their HEVs and LVs are crucial elements in those activities.

## Introduction

### Goals

Lymphoid and stromal cells accumulate and organize into tertiary lymphoid organs (TLOs) at ectopic sites in response to chronic inflammation in autoimmunity, microbial infection, graft rejection, and cancer where they assume structural and cellular characteristics of lymph nodes (LNs). High endothelial venules (HEVs) and lymphatic vessels (LVs) play key roles in LNs in transporting cells and antigens from and to the body. The questions to be addressed here are whether the HEVs and LVs in TLOs function and are regulated in a manner similar to those in LNs.

### Background

My research group became intrigued by the concept of TLOs in the course of two apparently unrelated series of investigations. The first was the characterization of mice that were transgenic for a construct of the rat insulin promoter driving expression of lymphotoxin alpha (LTα) (1) (in those days known as TNFβ, despite having been described as LT previous to the discovery of TNF). We made the rat insulin promoter lymphotoxin (RIPLT) mouse in order to develop a model of type 1 diabetes, since we knew that LT could induce inflammation. The transgene was not only expressed in the β cells in the islets of Langerhans in the pancreas as expected but also in the kidney and skin, most likely because the entire promoter with its negative regulatory elements was not included in the construct. At all sites of transgene expression, lymphoid cells accumulated, which were organized into distinct T and B cell areas (“compartmentalization”). Despite several attempts to drive the animals to β cell destruction and diabetes, the mice were healthy (2) unless a costimulator molecule such as B7-1 was also expressed in the β cells. Thus, the model resembled the early peri insulitis and non-destructive insulitis of diabetes. At the same time, we were collaborating with David Chaplin on the LTα knock out mouse that has no LNs (3). We realized that the consequence of ectopic expression of LT in the RIPLT mouse was the production of organized infiltrates that resembled LNs. We called them TLOs (4), a term that had been previously used to designate any lymphoid infiltrate (5). The process by which TLOs arise and organize was designated as lymphoid neogenesis (4).

In later years, I became especially interested in the vasculature of TLOs as I realized that understanding how cells enter into TLOs would provide insight into this accumulation and would indicate whether or not the apparent organization reflected function. That is, the presence of HEVs might indicate that naïve cells could enter the TLO, and the presence of LVs could indicate a method of entrance of antigen-presenting cells, thus providing in a single location, the elements to generate an immune response. This manuscript addresses these questions.

## Tertiary Lymphoid Organs

### Characteristics

Tertiary lymphoid organs, which have been described in almost every organ of the body, are also known as tertiary lymphoid structures, ectopic lymphoid tissues, or tertiary lymphoid tissues. They are distinguished from primary lymphoid organs – the thymus and bone marrow, and secondary lymphoid organs (SLOs) – the LNs, spleen, and Peyer’s patches, in that they arise in response to inflammation or inflammatory cues, rather than in ontogeny and are ectopic to canonical lymphoid organs. They usually do not have a capsule but are rather contained within the confines of another organ.

**Figure 1:**
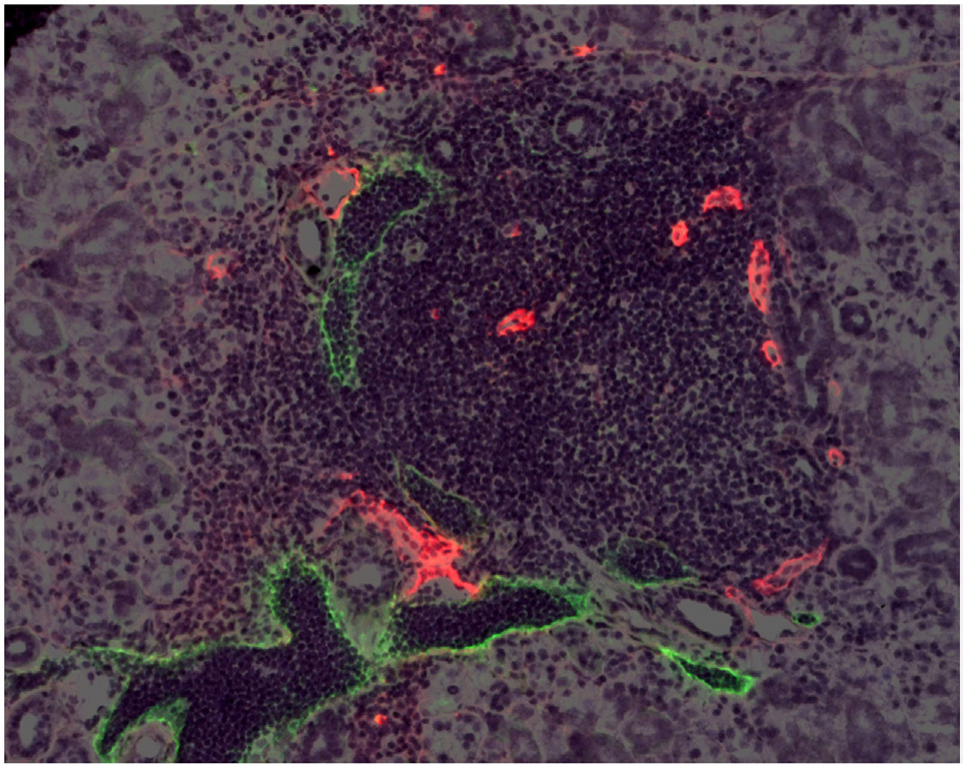
**High endothelial venules and lymphatic vessels in a TLO**. A mouse salivary gland TLO. HEVs are stained red with an antibody to MECA-79. LVs are stained green with an antibody to LYVE-1. From “Transgenic LacZ under control of Hec-6ST regulatory sequences recapitulates endogenous gene expression on high endothelial venules” by Liao et al. (11). Copyright (2007) National Academy of Sciences, USA.

Tertiary lymphoid organs are similar to LNs (6) with regard to their cellular content, stromal components, lymphoid chemokines (7), vasculature, and organization. Cells include compartmentalized T and B cells and antigen-presenting cells, including follicular dendritic cells and dendritic cells. CD8 and CD4 subsets include naïve, Treg, and T follicular helper cells (8, 9). B cells may be organized into germinal centers with plasma cells. HEVs (10), LVs (11, 12) (Figure 1), and conduits with fibroblastic reticular cells (13), all components of LNs, have also been described. In LNs, CCL19 and CCL21 direct T cells and DCs to the paracortical region, and CXCL13 directs B cells to the B cell follicles. These chemokines and cells that express their receptors are also expressed in TLOs (7). TLOs can be distinguished from acute inflammation; they generally include few granulocytes, and they are not necessarily destructive, although they may transform into tissue damaging entities. The plasticity of TLOs is seen in the case of the infiltrates in the pancreas in type 1 diabetes in the NOD mouse. Initially, the cellular infiltrates are disorganized and lack HEVs; then the infiltrates assume the characteristics of TLOs, with T and B compartmentalization and HEVs and LVs (14, 15); later, the lymphoid cells become activated, β cells are destroyed, and eventually the inflammation and thus, the TLO, is resolved as antigen is eliminated.

Tertiary lymphoid organs differ from LNs in that they generally do not have a capsule, they are not confined to a fixed location in the body, they develop postnatally, and as noted above, they exhibit plasticity. This is not to say that LNs do not respond to their environment; they most certainly do with proliferation and changes in vasculature and cell and antigen accessibility in the course of inflammation [see, e.g., Ref. (16)].

### Functions

Tertiary lymphoid organ functions vary depending on the location, stimulus and kinetics of inflammation, and cellular activation. The strongest evidence that TLOs are harmful in exacerbating autoimmune disease derives from studies in rheumatoid arthritis. In some patients, evidence that somatic mutation and affinity maturation occur in the locus of the TLO in the joint provides support for a harmful role leading to determinant spreading. On the other hand, the presence of Tregs in some TLOs (17) suggests that they can play a beneficial role by limiting inflammation. Additional evidence for a beneficial role is provided from several clinical studies of cancer, which indicate that the presence of TLOs in tumors in breast, colon, or lung predicts a favorable outcome, suggesting that the TLO site provides a locus for antigen activation and destruction of tumor, reducing dissemination of the malignant cells through the body (18). Nevertheless, Tregs in tumor TLOs can act as brakes on their defensive role (19, 20).

## HEVs: Characteristics, Functions, and Regulation in TLOs

### Characteristics

The presence of HEVs could be considered an essential trait distinguishing TLOs from acute inflammation. The endothelial cells in postcapillary venules in TLOs, as in LNs, tonsils, and Peyer’s patches, exhibit a typical cuboidal appearance. LN HEVs express a particular set of genes that facilitate their interactions with blood stream naïve and central memory cells that result in rolling, firm adhesion, and transmigration from the vessel into the parenchyma. HEVs in TLOs express the same molecules: CCL21 (7), ICAM-1 (4), and peripheral and/or mucosal addressins, PNAd (10) and MAdCAM-1 (4). Expression of these proteins allows the egress from the blood stream into the parenchyma of LNs of cells of the naïve and central memory phenotype that express CCR7, LFA-1, L-selectin (CD62L), and α4β7.

### Functions

The evidence is quite strong that HEVs in TLOs function similarly to those in LNs, allowing naïve and central memory cells to leave the blood stream and enter into the parenchyma of the tissue where they can interact with their cognate antigen. First, as noted above, they express the molecules that allow naïve and central memory cells to interact. Second, cells expressing CCR7, LFA-1, L-selectin (CD62L), and α4β7, the ligands for the receptors on HEVs, are found in TLOs. Third, several instances of T cell activation and memory generation occurring directly in the TLO have been described. These include generation of memory cells for graft rejection in skin TLOs (21) and presentation and activation of Teffector or Treg cells (19, 22). *In vivo* imaging of the transit of naïve cells into TLOs and their interaction with antigen-presenting cells will solidify the conclusion that HEVs function similarly in LNs and TLOs, and that HEVs in TLOs are the sites of entrance of naïve cells to undergo activation and differentiation and generation of memory cells.

### Regulation

High endothelial venules are regulated similarly in TLOs and SLOs. LTα alone induces MAdCAM-1 in endothelial cells *in vitro* (23, 24), *in vivo* in mesenteric LN HEVs (16), and in HEVs in TLOs (23) through TNFR1 (25). Abluminal PNAd in LN HEVs is generated through modification of a variety of glycoproteins. These modifications include sulfation, which is essential for PNAd (also called L-selectin ligand) interaction with its receptor, L-selectin (CD62L) that is expressed on the surface of naïve and central memory lymphocytes. Sulfation is induced in peripheral LN HEVs by sulfotransferases (26, 27). LTαβ regulates the HEV sulfotransferase in both LNs (16, 28) and TLOs (10) through the alternative NFκB pathway (29).

## LVs: Characteristics, Functions, and Regulation in TLOs

### Characteristics

Lymphatic vessels play key roles in the body in fluid and lipid balance. They are crucial in the immune system in providing communication of the lymphoid organs with the rest of the body. Lymphatic capillaries are thin-walled, blind-ended vessels that express CCL21, LYVE-1, PROX-1, podoplanin, VEGFR-2, and VEGFR-3 and are the initial entry point into LNs from the tissues for antigen and antigen-presenting cells. The endothelial cells on the tips of lymphatic capillaries are most frequently in a zipper-like arrangement (30). They connect to collecting vessels whose cells exhibit a button-like arrangement that are usually low or negative for LYVE-1, but do express PROX-1. The latter is especially highly expressed in valves that are characteristic of collecting vessels. A layer of smooth muscle cells surrounding collecting vessels contributes to their pumping action. Afferent collecting vessels carry substances to LNs, whereas efferent vessels allow egress of activated cells from the LN into the next LN in the chain and eventually into the blood stream *via* the right or left subclavian veins. In addition to serving as routes of fluid, lipid, cell, and cytokine transport, recent publications attest to the ability of LN LVs to present self or foreign antigens, either directly or by transfer to antigen-presenting cells (31–34).

Thin-walled vessels that are positive for lymphatic markers, including LYVE-1, PROX-1, podoplanin in mouse and human or D2-40 in human have been noted in many TLOs [summarized in Ref. (12)]. These include chronic kidney rejection (35, 36), cardiac allografts (37), transgenic mouse models (38), age-related Sjögren’s-like disease in the mouse (11), and a transgenic model of primary Sjögren’s in the mouse (Truman et al., in preparation). Confusingly, a *reduced* number of LVs in kidneys of mouse strains with a higher preponderance of spontaneous kidney TLOs have been noted (39). However, the latter report did not indicate the actual location of the LVs (i.e., in the vicinity or not of the TLO). CCL21-expressing TLO-associated vessels have been described in rheumatoid arthritis, Crohn’s disease, Sjögren’s syndrome, chronic allograft rejection (40), and pancreatic infiltrates in NOD (15) and RIPLTα mice (7). Nevertheless, much still needs to be learned. Collecting vessels with valves and smooth muscle cells neither have been specifically identified entering or leaving TLOs nor have the vessel walls been characterized with regard to their zipper or button-like morphology.

### Functions

Do the LVs in TLOs carry out the same functions as those in LNs? It is likely that they contribute to fluid drainage, although this has not been carefully analyzed. Do LVs carry antigen and cells to TLOs and cells away from TLOs, as do afferent and efferent vessels in LNs? TLO LVs frequently contain cells (11, 39), supporting the concept that they act as transporters as does their expression of CCL21 indicating they interact with CCR7-expressing cells. However, the fact that LVs in some TLOs appear to be packed with cells suggests that there could be a defect in cellular drainage and that their efferent function is compromised. Sphingosine-1 phosphate (S1P) is expressed in lymph and downregulates its receptor (S1P1) on lymphocytes. Lymphocytes in LNs reexpress the receptor and migrate toward the S1P in lymph and egress from the LN. FTY720 (fingolimod) is an agent that is used in transplantation and multiple sclerosis treatment that acts as an agonist for the receptor, causing its internalization resulting in lymphocyte accumulation in LNs (41), thus acting as an immunosuppressant. When NOD mice with pancreatic TLOs are treated with this agent, they are protected from islet destruction and diabetes, consistent with the concept that their LVs carry out an efferent function (42). In our hands, this treatment inhibits disease only at the time that the mice exhibit TLOs (15), although others have determined that FTY720 treatment is partially effective even after the development of elevated blood sugar (43). The pancreatic TLOs exhibit an increased insulitis score after FTY720 treatment, indicating that cells are trapped in these structures. Within days of cessation of drug treatment, islet destruction and diabetes occurs (15, 42). These data are consistent with the concept that the S1P gradient affects lymphocyte trafficking in TLO LVs. Further supporting the concept that the FTY720 effects are at least partially due to an effect on the TLOs is the observation that FTY720 treatment inhibits cellular migration from inflamed tissues into afferent LVs (44, 45). It must be noted that FTY720 treatment is also most likely affecting trafficking from LNs in this context, complicating interpretation of the data. This needs to be evaluated in situations where the events in TLOs can be isolated from LNs, as was done in a previous transplantation model (21). A straightforward test of these conclusions would be to determine if LVs in TLOs produce S1P as they do in LNs (46). If so, systemic inhibitors of lymphocyte trafficking may function directly at the TLO site by preventing traffic to the LNs from the TLO, a potential site of self antigen presentation.

Lymphatic vessels transport soluble or cell-associated antigens into LNs. Recently, it has become apparent that plasmalemma vesicle-associated protein (PLVAP), visualized by reactivity with the MECA-32 antibody, heretofore considered limited to blood vessels, is also expressed on the lymphatic endothelial cells in the lymphatic sinus in the LN. PLVAP positive lymphatic endothelial cells contribute to sieving of lymphocytes and high molecular weight antigens entering the LN *via* the conduits (47). Since TLOs include conduits (13), it seems reasonable to ask whether LVs in TLOs perform antigen and cell transport and sieving functions similar to those in LNs. Antigen transport may be less important than in SLOs because the antigen is an actual component of the TLO. As long as antigen-presenting cells are in the TLO (as they usually are), the issue is moot. Proteins such as insulin in the pancreatic islet are in immediate proximity or, as constituents of β cells, even contribute to the structure of the TLO in type 1 diabetes. With regard to the sieving function, an analysis of expression of PLVAP in TLOs by co-staining with MECA-32 and LYVE-1 or PROX-1 should be fairly straightforward. Functional analysis by crossing PLVAP-deficient mice to mice with TLOs or MECA-32 inhibition of migration of cells or labeled antigen to TLOs could address the function of LVs in TLOs. As noted above, LVs in LNs present self antigens (31–34), either directly through their expression of MHC molecules or by passing antigen on to “classical” antigen-presenting cells. Such presentation of self antigen by LVs (31) could be a way to induce either tolerance or T cell activation in LNs or in TLOs. The ability of TLO LVs to present antigen to induce either of these outcomes has not been investigated. Tregs are found in tumor TLOs and can inhibit cytotoxic T cells from attacking the tumor (19), indicating that understanding the mechanisms of self and tumor presentation to both potential effector T cells and Tregs is crucial to our ability to harness TLOs for both prophylaxis and therapy of cancer and autoimmune diseases.

### Regulation

The most commonly accepted scenario for the development of LVs in ontogeny is that they sprout from veins (48) under the influence of SOX18, PROX-1, growth factors and their receptors (VEGF-C and VEGF-D and VEGFR-2 and VEGFR-3), and platelets (49) [reviewed in Ref. (50)]. Although the evidence is quite strong for this mechanism in the case of the LVs sprouting from the cardinal vein, it has become apparent that the situation is somewhat more complex. The first indication that additional mechanisms of lymphangiogenesis existed was the discovery of lymphangioblasts that could be distinguished from blood endothelial cells, in developing animals as distinct as tadpoles (51), chickens (52), and mice (53–55). Several recent studies have revealed that the origin of LVs is quite heterogeneous. Mahadevan et al. reported that LVs in the intestine are derived from arteries, rather than veins (56); Stanczuk et al. described hemangiogenic precursors that contribute to mesenteric LVs (57); Martinez-Corral et al. described the non-venous origin of dermal LVs in a process these authors termed lymphvasculogenesis (58); Klotz et al. also described a non-venous origin of cardiac LVs (59); and Nicenboim et al. reported that LVs derive from angioblasts in zebra fish (60).

Given the rapidly emerging data regarding the heterogeneity and the likelihood of organ-specific regulation of lymphangiogenesis in ontogeny (61), it becomes more important, and perhaps even more daunting, to understand the regulation of lymphangiogenesis in inflammation, particularly in chronic inflammation in TLOs. Do LVs in TLOs arise from veins? The presence of angiogenesis and platelets in inflammation supports such a scenario, as does the existence of vessels that express both HEV and LV markers in the inflamed LN (16). On the other hand, host-derived bone marrow precursors have been noted in association with LVs in the TLOs of chronically rejecting kidneys (36) suggesting a non-venous origin. Lymphangiogenesis in inflammation could occur by sprouting from existing LVs. But what cells orchestrate these events? DCs, macrophages, T and B cells have been implicated in the regulation of LVs in acute inflammation (16, 62–64), but different cells may be important at different times in different tissues. For example, B cells appear to be important in stimulating lymphangiogenesis that occurs in LNs during inflammation, but only at the early stages after immunization (16, 62) suggesting that they may be of lesser importance in chronic inflammation in TLOs. The participation of macrophages in lymphangiogenesis in acute inflammation has been documented, although the precise nature of their role is controversial. Various possibilities include integration into LVs, trans differentiation into lymphatic endothelial cells (65), and provision of growth factors [summarized in Ref. (66)] and cytokines. The expression of LYVE-1 by macrophages is supportive evidence for the former possibility; on the other hand, the expression of this marker on both macrophages and LECs may be serendipitous.

Several studies have evaluated the negative and positive roles of cytokines in lymphangiogenesis, although the bulk of these studies have evaluated acute inflammation rather than TLOs. There have been reports of negative regulation of lymphangiogenesis by IFNγ (67) and TH2 cytokines IL-4 and IL-13 (68) and positive regulation by IL-17 (69), LTα, and TNF (38, 70). LT is crucial for both lymphoid organ development and TLOs, and LTα_3_ contributes to lymphangiogenesis in development (38). LVs are apparent in RIPLT TLOs even in the absence of LTβ and before extensive cellular infiltration, suggesting a direct activity of the cytokine (38). On the other hand, LVs are inhibited by treatment with a LTβR–Ig in a CXCL13-induced model of a thyroid TLO (71). Further analysis of lymphangiogenesis in spontaneous TLOs, such as Sjögren’s syndrome, rheumatoid arthritis, and type 1 diabetes, may reveal which cytokines regulate this process. Additional studies *in vivo* and *in vitro* should reveal the mechanism of cytokines’ regulation as direct effects on lymphatic endothelial cells and/or as indirect effects through the facilitation of lymphatic growth factor producing cells.

Recent research reveals LV plasticity in gene function and regulation. It is obvious that their different environments (mesentery, skin, etc.) influence their gene expression. Inflammation in these diverse locales also contributes to changes in cytokine and growth factor expression. TNF and oxazolone treatment induce higher levels of CCL21 on dermal LVs, and presumably enhance cellular migration (72). Immunofluorescence and microarray studies revealed an increase in several additional inflammatory genes (73), although some genes, including *VEGFR-3 and PROX-1*, are downregulated. As yet, no such comparisons have included TLO LECs, which would be of particular interest because of the chronic nature of stimulation. Recently described methods to isolate LVs by virtue of their transgenically induced expression of a tomato red fluorescent protein should allow direct comparison of gene expression and function of LVs from different sites and acute and chronic inflammation (74, 75) and provide precise characterization of TLO LVs.

## Concluding Remarks

In this communication, I have provided background from a personal perspective of the development of the TLO field, and more particularly the role of the vasculature present and employed in TLOs. Although questions remain concerning the precise functions of HEVs and LVs in TLOs, the evidence is quite strong that they do behave as they do in LNs. The appropriate experimental tools (*in vivo* imaging, mice with fluorescent HEVs and LVs) are available to address these issues. The answers to these questions will provide insight, not only into TLOs but also into processes of antigen presentation in LNs and tissue destruction in acute inflammation. This is turn will provide understanding and methods to induce or inhibit TLOs in autoimmunity, microbial infection, organ rejection, and cancer.

## Author Contributions

The author confirms being the sole contributor of this work and approved it for publication.

## Conflict of Interest Statement

The author declares that the research was conducted in the absence of any commercial or financial relationships that could be construed as a potential conflict of interest.

## Abbreviations

HEV, high endothelial venule; LEC, lymphatic endothelial cell; LN, lymph node; LT, lymphotoxin; LV, lymphatic vessel; SLO, secondary lymphoid organ; TLO, tertiary lymphoid organ.
